# Advancing the Metabolic Dysfunction-Associated Steatotic Liver Disease Proteome: A Post-Translational Outlook

**DOI:** 10.3390/genes16030334

**Published:** 2025-03-12

**Authors:** Kushan Chowdhury, Debajyoti Das, Menghao Huang

**Affiliations:** 1 Department of Medicine, Vatche and Tamar Manoukian Division of Digestive Diseases, University of California Los Angeles, Los Angeles, CA 90095, USA; kchowdhury@mednet.ucla.edu (K.C.); ddas@mednet.ucla.edu (D.D.); 2 Department of Biochemistry and Molecular Biology, Indiana University School of Medicine, Indiana University, Indianapolis, IN 46202, USA; 3 Center for Diabetes and Metabolic Diseases, Indiana University School of Medicine, Indianapolis, IN 46202, USA; 4 Melvin & Bren Simon Comprehensive Cancer Center, Indiana University School of Medicine, Indianapolis, IN 46202, USA

**Keywords:** liver, post-translational modifications, MASLD, MASH, proteomics

## Abstract

Metabolic dysfunction-associated steatotic liver disease (MASLD) is a prevalent liver disorder with limited treatment options. This review explores the role of post-translational modifications (PTMs) in MASLD pathogenesis, highlighting their potential as therapeutic targets. We discuss the impact of PTMs, including their phosphorylation, ubiquitylation, acetylation, and glycosylation, on key proteins involved in MASLD, drawing on studies that use both human subjects and animal models. These modifications influence various cellular processes, such as lipid metabolism, inflammation, and fibrosis, contributing to disease progression. Understanding the intricate PTM network in MASLD offers the potential for developing novel therapeutic strategies that target specific PTMs to modulate protein function and alleviate disease pathology. Further research is needed to fully elucidate the complexity of PTMs in MASLD and translate these findings into effective clinical applications.

## 1. Introduction

**Figure 1:**
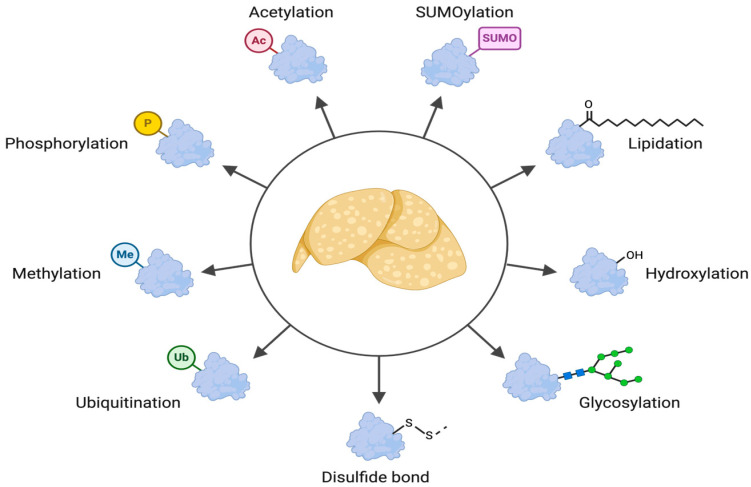
**Overview of PTMs and their impact on MASLD.** This illustration provides a visual summary of the main PTMs discussed in this review and their effects on different aspects of MASLD pathology. It highlights the complexity and crosstalk of these modifications, emphasizing their crucial role in disease progression.

Advancing our understanding of metabolic dysfunction-associated steatotic liver disease (MASLD) is critical due to its increasing prevalence and strong association with metabolic disorders such as obesity [1] and diabetes [2,3]. This complex condition is characterized by the accumulation of lipids in the liver, which can lead to inflammation, fibrosis, and potentially cirrhosis [4]. Recent research has emphasized the pivotal role of post-translational modifications (PTMs) in the pathogenesis of MASLD, highlighting how alterations in protein function through modifications such as phosphorylation, ubiquitylation, acetylation, and glycosylation can influence disease progression and treatment outcomes (Figure 1). Post-translational modifications (PTMs) have emerged as key regulators in the pathogenesis of MASLD, influencing protein stability, localization, and activity. The use of advanced proteomic techniques, including mass spectrometry, has significantly advanced the identification of differentially abundant proteins in MASLD, highlighting potential biomarkers of disease severity [5,6,7]. Furthermore, insights gained from differential gene expression studies [8,9] and multi-omics approaches [10,11] have unveiled significant key pathways related to lipid metabolism and hepatic dysfunction, paving the way for targeted therapeutic strategies. Despite these advancements, the field faces ongoing challenges, including the absence of universally accepted pharmacological therapies for MASLD and the inherent complexity of the disease’s multi-faceted nature. The variability in patient responses necessitates personalized treatment approaches that consider genetic, ethnic, and phenotypic differences, which are vital for effective clinical interventions. As research continues to unveil the intricacies of MASLD through proteomic and PTM-focused insights, the hope for developing effective biomarkers and therapies remains strong, addressing an urgent need in liver disease management.

This review aims to systematically explore the roles of phosphorylation, ubiquitylation, acetylation, and glycosylation in MASLD progression. By integrating evidence from proteomic, genomic, and metabolomic studies, we highlight PTMs as potential therapeutic targets and discuss their implications in disease pathogenesis. Furthermore, we explore emerging technologies, including artificial intelligence and multi-omics approaches, used in advancing MASLD research. By providing a comprehensive analysis of PTM-mediated mechanisms, this review seeks to bridge the gap between basic molecular research and clinical applications, offering insights for future therapeutic interventions.

## 2. Advances in Proteomic Technologies

Recent advancements in proteomic technologies have significantly enhanced our understanding of liver diseases including MASLD, metabolic dysfunction-associated steatohepatitis (MASH), cirrhosis, and hepatocellular carcinoma (HCC). Techniques such as mass spectrometry, particularly in the context of PTMs, have facilitated the identification of specific protein alterations associated with these conditions. The implementation of top-down proteomics allows for the characterization of proteoforms—distinct versions of proteins arising from gene variations, RNA splicing, and post-translational processing—which provides a more comprehensive and nuanced view of liver disease progression compared to traditional bottom-up approaches.

In a study analyzing plasma samples from 158 histologically characterized MASLD patients, a total of 72 proteins were found to be differentially abundant between individuals with early-stage fibrosis (F0-2) and those with advanced fibrosis (F3-4). Notably, two proteins—Insulin-like growth factor-binding protein complex acid labile subunit (ALS) and Galectin-3-binding protein (Gal-3BP)—were highlighted for their discriminatory performance [12]. ALS plays a crucial role in modulating the insulin-like growth factor (IGF) axis, which regulates cell proliferation, differentiation, and metabolism. Dysregulation of the IGF pathway has been linked to hepatic steatosis, fibrosis, and hepatocarcinogenesis. Elevated ALS levels in MASLD patients with advanced fibrosis suggest a potential compensatory response to altered IGF signaling, contributing to extracellular matrix deposition and fibrotic progression.

Gal-3BP is involved in immune regulation, inflammation, and extracellular matrix remodeling. It interacts with galectins and integrins, modulating cell adhesion and immune cell recruitment. In MASLD, Gal-3BP has been associated with heightened inflammatory signaling and profibrogenic activity, linking it to liver fibrosis progression. Its elevated expression in advanced fibrosis may reflect increased hepatic immune activation and fibrotic matrix turnover.

The validation of these proteins in an independent cohort resulted in an area under the receiver operating characteristic curve (AUROC) of 0.796, indicating their robust ability to assess advanced liver fibrosis compared to traditional non-invasive fibrosis assessment models [12]. Compared to traditional non-invasive fibrosis assessment models such as the Fibrosis-4 (FIB-4) index [13] and the NAFLD Fibrosis Score (NFS) [14], which typically have AUROC values in the range of 0.70–0.80, ALS and Gal-3BP demonstrate comparable, if not superior, diagnostic potential. Unlike existing serum-based models that rely on standard biochemical parameters, these proteins offer a more mechanistically driven approach, with potentially improved specificity for MASLD-related fibrosis. However, further large-scale validation studies are necessary to determine their clinical utility and integration into existing diagnostic frameworks.

The exploration of genetic influences on proteomic outcomes has been further elucidated using Multi-marker Analysis of GenoMic Annotation (MAGMA) and Transcriptome-Wide Association Studies (TWASs). These approaches enable the imputation of gene expression values using liver-specific data models, capturing cis-acting genetic effects on gene expression. The analysis revealed significant pathways related to glycogen biosynthesis, exocytosis, and activin receptor activity, which are known to play roles in MASLD pathogenesis. These pathways are intricately linked to PTMs, as glycosylation and acetylation can influence glycogen biosynthesis, ubiquitylation regulates exocytosis, and phosphorylation modulates activin receptor activity. Such insights provide a more comprehensive view of the molecular underpinnings of MASLD, offering valuable insights for the identification of therapeutic targets and diagnostic markers.

## 3. Exploring Liver Disease Pathogenesis: The Pursuit of Biomarker Discovery

Biomarkers are measurable molecules found in biological samples, such as blood or tissue, that indicate the presence or severity of a disease. In the context of MASLD, biomarkers are essential for diagnosis, monitoring disease progression, and predicting treatment responses.

### 3.1. Proteomics-Based Biomarker Studies

One study evaluated proteomics-based biomarkers in both plasma and liver tissue collected from patients during liver biopsies performed to diagnose MASLD. An untargeted proteomics analysis was performed on samples from 64 patients. The researchers identified twenty plasma proteins that were either upregulated (increased) or downregulated (decreased) in patients with MASH, a type of MASLD characterized by inflammation and liver cell damage, compared to those without MASH [15]. While the study does not disclose the specific names of these proteins, this finding suggests the potential for using these proteins as biomarkers for diagnosing and monitoring MASH. However, further research is needed to validate these findings and determine their clinical applications.

In another study, researchers sought to identify new protein targets within the causal pathway of MASLD. A comprehensive analysis of 2941 plasma proteins revealed that 1022 (34.8%) were associated with MASLD after adjusting for multiple testing [16]. Further analysis, which included adjusting for lifestyle factors, including smoking status, number of pack-years of smoking, grams of alcohol consumed per week, education, and physical activity, narrowed down the number of proteins associated with MASLD to 929. To determine causality, researchers applied Mendelian randomization (MR) analysis, which uses genetic variants as instruments to explore the causal relationships between exposures and outcomes. In this case, it helped to identify the proteins that are potentially involved in the causal pathway of MASLD, offering promising targets for therapeutic development.

While Mendelian randomization (MR) analysis provides a powerful tool for inferring causal relationships between plasma proteins and MASLD, it is not without limitations. One key concern is the potential for pleiotropy, where genetic variants used as instrumental variables influence multiple traits beyond the intended exposure. This could introduce bias and lead to the misinterpretation of causal relationships. Future studies should incorporate robust sensitivity analyses, such as MR–Egger regression and weighted median approaches, to mitigate pleiotropic effects.

Despite these comprehensive adjustments, the potential for residual confounding remains, as lifestyle behaviors are complex and influenced by multiple unmeasured factors. Moreover, approaches to multiple testing correction using the Matrix Spectral Decomposition (MSD) method should be critically evaluated to ensure robust statistical control. Future research should provide greater transparency on these statistical adjustments to enhance the reproducibility and confidence in these findings.

### 3.2. Genetic Variants and PTMs in MASLD

Genetic variants can influence PTMs and contribute to MASLD pathogenesis. Single-nucleotide polymorphisms (SNPs) in genes encoding kinases, phosphatases, or their substrates can alter enzyme activity or substrate availability, leading to dysregulated PTMs and downstream effects on cellular processes. For example, SNPs in the AMPK gene have been associated with altered AMPK activity and susceptibility to MASLD. Similarly, SNPs in genes encoding proteins involved in ubiquitylation or glycosylation can affect PTMs and contribute to disease progression. A recent study investigated the impact of genetic variants on MASLD and identified several SNPs associated with disease progression [17]. These SNPs were located in genes involved in various pathways, including lipid metabolism, inflammation, and fibrosis. The study also found that some of these SNPs were associated with the altered expression or activity of enzymes involved in PTMs.

For example, an SNP in the *PNPLA3* gene (rs738409 C > G p.1148M variant) was associated with the decreased phosphorylation of AMPK, a key regulator of lipid metabolism. As anticipated, SNPs associated with associated aspects of lipid handling in MASLD were also highlighted—rs72613567 in the hydroxysteroid 17-β dehydrogenase 13 (*HSD17B13*) gene (lipid metabolism), rs58542926 in the transmembrane 6 superfamily member 2 (*TM6SF2*) gene (lipid transport), and rs641738 in the membrane-bound O-acyltransferase domain containing 7 (*MBOAT7*) gene (lipid synthesis).

These findings suggest that genetic variants can influence PTMs and contribute to MASLD pathogenesis. Further research is needed to fully elucidate the role of genetic variants in PTM regulation and MASLD development.

### 3.3. Proteomic Changes in Animal Models

A study using a mouse model of MASLD investigated proteomic changes in the livers of mice fed a high-fat diet (HFD) to induce MASLD. The researchers first divided the mice into three groups: one group was fed a standard chow diet (CON), another group was fed an HFD, and the third group was fed an HFD but also had an exercise regimen (HFX). They then used two-dimensional gel electrophoresis to analyze protein expressions in the livers of these mice. This technique separates proteins based on their charge and size, allowing for the visualization and quantification of different proteins. The study identified 31 protein spots that were increased and 27 that were decreased in the HFD group compared to the control group. This indicates that an HFD can significantly alter protein expression in the liver, potentially contributing to the development of MASLD. However, while animal models provide valuable insights into MASLD pathogenesis, it is essential to acknowledge their limitations. Mice and humans exhibit significant differences in their metabolism and immune systems, which can influence the applicability of animal model findings to human subjects. Therefore, human liver proteomic studies are necessary to shed light on the potential for extrapolation from animal model studies.

A comprehensive study of proteomic changes in the livers of human subjects with NASH compared to healthy controls was recently conducted. The high-throughput proteomic analysis of liver biopsies identified 1644 differentially abundant proteins between the groups. Notably, two-thirds of these proteins showed an increased abundance in NASH, and their functions were primarily related to extracellular matrix remodeling, signal transduction, and immune response. The remaining third, which had a decreased abundance in NASH, were predominantly involved in metabolic processes, such as fatty acid and ethanol/drug metabolism. These findings demonstrate that NASH induces substantial alterations in liver protein expression, similar to those observed in animal models, and provide further evidence of the role of proteomic changes in MASLD development [18].

### 3.4. The Role of Artificial Intelligence and Multi-Omics Approaches

The advent of artificial intelligence (AI) has transformed analytical methods, advancing diagnosis, personalized treatments, and therapeutic strategies for liver diseases, including MASLD [19]. Proteomics and other omics approaches (such as metabolomics, which studies small molecules involved in metabolism, and the study of the methylome, which focuses on the patterns of DNA methylation) provide novel insights into liver disease.

While transcriptomic (RNA transcript profiling) and genomic (gene-level analysis) studies are more prevalent in liver disease research, proteomics and other levels of omics remain relatively under-explored. Proteomics is particularly valuable as it directly measures the proteins present and active in cells and tissues, offering a more functional perspective than gene expression alone. Increased research in these under-explored areas could lead to a deeper understanding of liver disease mechanisms and the identification of new therapeutic targets.

## 4. Post-Translational Modifications and MASLD

Herein, we present a comprehensive list of the most potent and thoroughly investigated post-translational modifications (PTMs) of proteins, highlighting their significant roles in either driving or protecting against MASLD and advanced liver diseases. These insights underscore the importance of PTMs in the progression of liver diseases and present potential opportunities for therapeutic intervention.

### 4.1. Phosphorylation

Phosphorylation modifies proteins by adding phosphate groups to serine, threonine, or tyrosine residues, acting as an on/off switch for signaling pathways (Table 1). Dysregulated phosphorylation plays a pivotal role in MASLD pathogenesis through mechanisms such as aberrant lipid metabolism, insulin resistance, mitochondrial dysfunction, oxidative stress, and inflammation [20,21]. For example, the phosphorylation of AMPK at the Thr172 site activates lipid oxidation and inhibits lipogenesis [22]. In contrast, phosphorylated NF-kB drives inflammatory cytokine production, worsening steatosis and fibrosis [23].

It is important to highlight that phosphorylation does not operate in isolation. It often interacts with other PTMs, such as ubiquitination and acetylation, to fine-tune protein function and cellular processes. For example, phosphorylation can create binding sites for E3 ubiquitin ligases, leading to the ubiquitination of proteins and their subsequent degradation. Similarly, phosphorylation can influence the acetylation status of proteins by modulating the activity of acetyltransferases and deacetylases. This intricate interplay between phosphorylation and other PTMs adds another layer of complexity to the regulation of MASLD, underscoring the importance of considering an integrated PTM network.

A. **MAPK Pathway Activation and Lipid Dysregulation**

Dysregulation of the mitogen-activated protein kinase (MAPK) pathway contributes significantly to MASLD pathogenesis. The hyperactivation of MKK6, a MAP2K component, triggers p38 phosphorylation, intensifying hepatic steatosis by increasing white adipose tissue (WAT) lipolysis and fatty acid influx into liver cells [24]. Similarly, c-Jun N-terminal kinases (JNK1 and JNK2) are pivotal in establishing liver steatosis and injury. The JNK1-mediated phosphorylation of IRS-1 at Ser307 results in insulin resistance (IR), while its activation promotes hepatocyte lipo-apoptosis [25,26,27]. These findings highlight the dual pro-steatotic and pro-apoptotic roles of MAPKs in MASLD.

B. **mTORC1 and Androgen Receptor (AR) Phosphorylation**

The mechanistic target of rapamycin complex 1 (mTORC1), a Ser/Thr kinase, mediates the phosphorylation of the androgen receptor (AR) at Ser96 [28]. This modification enhances the AR-driven transcription of lipogenic genes such as SREBP-1 and FASN, thereby promoting de novo lipogenesis and exacerbating liver steatosis, underscoring the intricate crosstalk between mTOR signaling and transcriptional regulation in hepatic lipid metabolism [4].

C. **Role of Protein Phosphatases in Steatosis**

The cellular phospho-proteomic status is dynamically regulated by protein phosphatases, which counteract kinase-mediated phosphorylation. Dysregulated phosphatase activity is emerging as a critical factor in MASLD pathogenesis.

The loss of functional hepatic phosphatase and tensin homolog (PTEN) [29] and T-cell protein tyrosine phosphatase (TCPTP) promote lipid accumulation [30]. PTEN and TCPTP have distinct substrate specificities, with PTEN primarily targeting phosphoinositide substrates while TCPTP dephosphorylates protein tyrosine residues. The loss of PTEN can lead to increased signaling through the PI3K/Akt pathway, promoting de novo lipogenesis and lipid accumulation. TCPTP deficiency can result in the increased tyrosine phosphorylation of proteins involved in insulin signaling, contributing to insulin resistance and hepatic steatosis, while elevated levels of protein phosphatases like PTP1B and protein phosphatase 2A (PP2A) drive steatosis through SREBP-1c and Sp1 activation [31]. Notably, the dephosphorylation of PP2A at Tyr307 enhances its activity [32,33], further stimulating hepatic steatosis. These findings suggest that the therapeutic targeting of phosphatases may offer novel avenues for MASLD management.

D. **AMPK-Mediated Lipid Regulation**

AMP-activated protein kinase (AMPK) serves as an energy sensor in hepatocytes, modulating lipid metabolism to counteract hepatic steatosis. AMPK activity is repressed during MASH and liver-specific knockout models of AMPK correlate with worsened liver damage in mice [34]. AMPK activation is protective in MASLD and occurs through the phosphorylation of Thr172 on its catalytic α-subunit, which enhances its activity. The phosphorylation of acetyl-CoA carboxylase (ACC) at Ser79 [35] by AMPK inhibits its activity, reducing malonyl-CoA synthesis and enhancing fatty acid oxidation. AMPK phosphorylation also promotes mitochondrial biogenesis by activating PGC-1α, a key regulator of mitochondrial gene expression. This leads to increased mitochondrial content and an improved fatty acid oxidation capacity, further contributing to the protective effects of AMPK in MASLD. Additionally, AMPK regulates downstream effectors such as sterol regulatory element-binding protein-1c (SREBP-1c) and fatty acid synthase (FASN), further attenuating lipid accumulation [36]. As MASLD progresses to MASH, the AMPK-mediated phosphorylation of proapoptotic caspase-6 at Ser257 inhibits its activity, thereby protecting hepatocytes from apoptosis [37].

E. **Lipophagy Regulation via VPS4A Phosphorylation**

Macrolipophagy, or simply lipophagy, serves as a defined mechanism wherein the autophagic machinery is utilized for the degradation of lipid droplets in organisms. During fasting conditions, the casein kinase 2 (CK2)-mediated phosphorylation of VPS4A in Ser95 and Ser97 residues stimulates lipophagy [38,39,40], which allows for the interaction of VPS4A with microtubule-associated protein 1 light chain 3 (LC3). It is crucial to note that VPS4A is not a core member of the autophagy machinery, and the absence of VPS4A did not influence key markers of autophagy activity, such as the quantity of LC3 puncta, LAMP2 vesicles, or the overall autophagy flux.

**Table 1:** Phosphorylation and its impact on MASLD.

PTM	Target Protein	PTM Detection Methodology	Mechanism of Action	Impact on MASLD	Reference
Phosphorylation	AMPK (Thr172)	Immunoblot	Activates lipid oxidation, inhibits lipogenesis, activates AMPK, inhibits ACC activity, reduces malonyl-CoA synthesis, enhances fatty acid oxidation	Ameliorates steatosis	[22]
	NF-κB/ p65	Immunoblot	Drives inflammatory cytokine production	Worsens steatosis and fibrosis	[23,41,42,43,44]
	p38 MAPK	Immunoblot	Intensifies hepatic steatosis by increasing WAT lipolysis and fatty acid influx	Worsens steatosis	[24]
	IRS-1 (Ser307)	Immunoblot	Induces insulin resistance	Worsens steatosis and insulin resistance	[25,26,27]
	AR (Ser96)	Immunoblot, Mass Spectrometry	Enhances AR-driven transcription of lipogenic genes	Worsens steatosis	[28]
	Caspase-6 (Ser257)	Immunoblot	Inhibits caspase-6 activity, protects hepatocytes from apoptosis	Ameliorates steatohepatitis	[36]
	VPS4A (Ser95, Ser97)	Phospho- proteomics/ Immunoblot	Stimulates lipophagy	Ameliorates steatosis	[37,38,39]
De- phosphorylation	PP2A (Tyr307)	Immunoblot	Enhances PP2A activity, stimulates SREBP-1c and Sp1 activation	Worsens steatosis	[31,32]

### 4.2. Ubiquitylation

Ubiquitylation is a post-translational modification orchestrated by a cascade of three enzymatic steps: activation by E1 ubiquitin-activating enzymes; conjugation by E2 ubiquitin-conjugating enzymes; and substrate attachment, mediated by E3 ubiquitin ligases [45]. The versatility of ubiquitylation arises from its ability to form diverse ubiquitin chains on substrates, determining whether their fate is proteasomal degradation, cellular trafficking, or signaling. The dysregulation of ubiquitylation machinery in MASLD leads to aberrant protein turnover, defective signaling, and heightened inflammation, contributing to disease progression. Ubiquitylation is the covalent attachment of ubiquitin, a 76-amino-acid protein, to substrate proteins that modulate a myriad of cellular processes, including proteostasis, signal transduction, and immune responses, all of which are implicated in MASLD pathogenesis (Table 2).

While ubiquitylation is crucial for protein regulation, the reverse process, deubiquitylation, is equally important. Deubiquitylases (DUBs) are enzymes that remove ubiquitin chains from proteins, counteracting the effects of ubiquitylation. DUBs play a critical role in maintaining protein homeostasis and regulating various cellular processes, including those involved in MASLD. The dysregulation of DUB activity can disrupt protein turnover, leading to the accumulation of misfolded proteins and cellular dysfunction. In the context of MASLD, altered DUB activity can contribute to disease progression by affecting lipid metabolism, inflammation, and fibrosis. Therefore, understanding the balanced interplay between ubiquitylation and deubiquitylation is essential for deciphering the complex mechanisms underlying MASLD.

A. **Lipid Metabolism**
  - **TRIM21:** Tripartite motif containing-21 (TRIM21), an E3 ubiquitin ligase, is induced by the combined effects of TNF-α and fatty acids in mice and human MASH livers. TRIM21 plays a protective role whereby it reduces hepatic lipogenesis and fructolysis by ubiquitinating and degrading de novo lipogenic players like SREBP1, ChREBP, A1CF, ACC, and FASN [46].
  - **Peroxisome Proliferator-Activated Receptor-α (PPARα):** PPARα is a key transcriptional factor central to fatty acid oxidation and ketogenesis. The ubiquitylation of PPARα by HUWE1 negatively regulates its activity. AdipoQ receptor 3 (PAQR3) directly interacts with PPARα, enhancing its polyubiquitination by increasing the interaction between PPARα and HUWE1 [45,47].
  - **ATP Citrate Lyase (ACLY):** ACLY catalyzes the conversion of citrate to acetyl-CoA, a precursor for lipid synthesis. Studies indicate that ubiquitylation at Lys540 governs ACLY’s enzymatic activity [48]. The inhibition of ACLY ubiquitylation increased lipogenesis in MASLD models, as demonstrated through metabolic flux analysis, further supporting its role in lipid homeostasis [49].
  - **SUMO and SUMO2:** These small ubiquitin-like modifiers regulate lipid metabolism. SUMOylation at Lys289 of the nuclear receptor liver receptor homolog 1 (LRH-1) is a protective mechanism against MASLD. LRH-1 K289R mice have an increased expression of oxysterol binding protein-like 3 (OSBPL3), which is a direct target gene of LRH-1. OSBPL3 promotes the post-translational activation of Srebp1c, thus promoting DNL [50].

Also, SREBP1c is sumoylated at Lys98 by the SUMO E3 ligase PIASy. This leads to the suppression of the hepatic lipogenic program upon fasting. PKA, activated by fasting signals, enhances this sumoylation and the interaction between SREBP1c and PIASy. Additionally, the PKA-mediated phosphorylation of SREBP1c further increases its sumoylation, ultimately leading to its degradation via ubiquitination [51].

B. **Inflammation and Immune Responses in MASLD**
  - **Nuclear Factor-κB (NF-κB) Pathway:** The NF-κB signaling pathway is tightly regulated by ubiquitylation. The ubiquitylation of IκBα at Lys21 and Lys22 targets this pathway for proteasomal degradation, leading to NF-κB activation and the subsequent transcription of pro-inflammatory genes [52]. Dysregulated ubiquitylation within this pathway potentially leads to chronic inflammation in MASLD, a key driver of MASLD progression.
  - **TRIM8 and MDM2:** TRIM8, an E3 ubiquitin ligase, regulates NF-κB signaling by ubiquitylating its pathway components, modulating inflammatory responses [53]. Additionally, the MDM2-mediated ubiquitylation of p53 was shown to suppress its pro-apoptotic functions, promoting cell survival under inflammatory conditions [54]. This suggests that altered MDM2 activity may contribute to MASLD by dampening p53-mediated apoptosis in response to cellular stress [55].

**Table 2:** Ubiquitylation and its impact on MASLD.

PTM	Target Protein	PTM Detection Methodology	Mechanism of Action	Impact on MASLD	Reference
Ubiquitylation	SREBP1, ChREBP, A1CF, ACC, FASN	Immuno- precipitation	Degradation of lipogenic proteins	Ameliorates steatosis	[46]
	PPARα	Immuno- precipitation	Degradation of PPARα	Worsens steatosis	[45,47]
	ACLY (Lys540)	Immuno- precipitation, Mass Spectrometry	Regulates ACLY activity	Worsens steatosis	[50,51]
	IRS-1 (Ser307)	Immunoblot	Induces insulin resistance	Impacts lipid metabolism	[25,26,27]
	IκBα	Immunoblot	Activates NF-κB, promotes inflammation	Worsens inflammation	[54]
	NF-κB pathway components	Immunoblot	Modulates inflammatory responses	Impacts inflammation	[47]
	P53	Immunoblot	Inhibits p53 activity, promotes cell survival	Worsens steatohepatitis	[48,49]
SUMOylation	LRH-1 (Lys289)	Immunoblot	Promotes OSBPL3 expression, activates SREBP1c	Worsens steatosis	[42]
	SREBP-1c (K=Lys98)	Immuno- precipitation	Promotes SREBP1c degradation	Ameliorates steatosis	[43]

### 4.3. Acetylation

Acetylation is a critical PTM regulating diverse cellular processes, including transcription, metabolism, and signal transduction. This modification typically involves the transfer of an acetyl group to lysine residues on target proteins, which is mediated by acetyltransferases (e.g., histone acetyltransferases, HATs) and removed by deacetylases (e.g., sirtuins and histone deacetylases, HDACs). Dysregulated acetylation disrupts cellular homeostasis and contributes to MASLD progression by altering lipid metabolism, inflammation, mitochondrial function, and fibrogenesis (Table 3).

Studies in MASLD mice models have shown a reduced acetylation of hepatic proteins and increased turnover rates of mitochondrial metabolic enzymes, suggesting reduced stability. Interestingly, the decreased acetylation of mitochondrial proteins may contribute to improved hepatic mitochondrial function in the early stages of MASLD, highlighting the dynamic nature of acetylation in response to a high-fat diet.

One way acetylation contributes to MASLD progression is through its impact on lipogenesis, the process of fatty acid synthesis. Increased adipose lipolysis and hepatic fatty acid uptake promote β-oxidation, which is linked to the PI3K/Akt pathway. Epigenetic regulators such as SNAIL1 and SLUG influence this process. SNAIL1 can bind to the promoters of lipogenic genes and recruit histone deacetylases HDAC1/2, leading to the deacetylation of histones H3K9 and H3K27, thereby suppressing lipogenesis and preventing excessive lipid accumulation in the liver. Conversely, SLUG interacts with the enzyme LSD1 to increase lipogenic gene transcription, potentially promoting lipid accumulation and MASLD development.

- **Acetylation in Lipid Metabolism**
  - **Sterol Regulatory Element-Binding Protein-1c (SREBP1c):** The acetylation of SREBP1c at Lys289 and Lys309 has been shown to stabilize the protein, promoting lipogenesis [56]. In diet-induced MASLD mice models, it was demonstrated that acetylation enhances SREBP1c’s DNA-binding affinity to lipid biosynthesis genes like FASN and ACC1, leading to triglyceride accumulation [57]. Chromatin immunoprecipitation (ChIP) assays confirmed the increased recruitment of acetylated SREBP1c to target promoters. Moreover, Sirtuin 6 (SIRT6), a nuclear enzyme with deacetylase, deacylase, and mono-ADP ribosyltransferase functions that utilize nicotineamide adenine dinucleotide (NAD+) to modify proteins has been implicated in maintaining metabolic homeostasis. SIRT6 represses SREBP1c transcriptional activity by deacetylating Lys289 [58].
  - **Liver X Receptor (LXR):** The acetylation of LXR, a nuclear receptor regulating lipogenesis, at Lys432 modulates its transcriptional activity [59]. LXR activity is induced by high cholesterol levels, activating lipogenic genes like SREBP1, FASN, ChREBP, and SCD1 [60,61,62].
  - **Cyclic AMP-responsive element-binding protein 3-like 3-hepatocyte-specific (CREBH):** The acetylation of CREBH by P300/CBP-associating factor (PCAF) at Lys294 is necessary for the function of its transcriptional activity to establish lipid homeostasis under fasting conditions [63].
  - **Carbohydrate response element binding protein (ChREBP):** ChREBP, a key regulator of glycolysis and lipogenesis, is acetylated at Lys672 by p300, leading to the increased transcription of lipogenic genes and hepatic lipid accumulation [64,65]. Studies using site-directed mutagenesis to prevent acetylation at this specific lysine residue have demonstrated the crucial role of ChREBP acetylation in regulating lipogenesis [66].
  - **ATP-citrate lyase (ACLY):** The acetylation of ATP-citrate lyase at Lys540, Lys546, and Lys554 increases its stability and promotes lipid synthesis, contributing to hepatic steatosis [67]. Mass spectrometry analysis has been used to identify these specific acetylation sites and their impact on ACLY stability and activity.
  - **SLUG:** This transcription factor interacts with LSD1 to increase lipogenic gene transcription, potentially contributing to MASLD development [68]. Experiments involving gene knockdown and overexpression have shown the impact of SLUG on lipogenic gene expression and lipid accumulation in liver cells. LSD1 demethylates H3K9, leading to increased lipogenic gene transcription and potentially promoting lipid accumulation. Studies using LSD1 inhibitors have demonstrated the potential of targeting this enzyme to modulate lipogenesis and potentially alleviate MASLD progression [69].
- **Acetylation in Inflammation and Immune Responses**
  - **Nuclear Factor-κB (NF-κB):** The p65 subunit of NF-κB undergoes acetylation at Lys310, enhancing its transcriptional activity. In a study on the role of berberine (BBR), it was demonstrated that in macrophages, the global acetylation landscape of lipopolysaccharide (LPS)-induced protein acetylation is altered. BBR was shown to reduce the acetylation of the NF-κB subunit p65 at Lys310, leading to the inhibition of NF-κB translocation and transcriptional activity, ultimately suppressing the expression of inflammatory factors. BBR also combated the inflammatory response in acute LPS-stimulated mice by downregulating Lys310 acetylation in peritoneal macrophages. In obese mice, BBR alleviated metabolic disorder and inflammation while also causing a reduction in Lys310 acetylation in white adipose tissue [70].
  - **SIRT1 (Sirtuin 1):** SIRT1 deacetylates multiple targets involved in inflammation and metabolism. The loss of SIRT1 activity in liver-specific knockout mice resulted in the hyperacetylation of NF-κB p65, exacerbating hepatic inflammation [71].
- **Acetylation in Fibrosis and late-stage MASLD**
  - **Hippo Pathway Effectors YAP and TAZ:** Yes-associated protein (YAP) and its paralog transcriptional coactivator with PDZ-binding motif (TAZ or WWTR1) are hyperacetylated during hepatic fibrosis in HSCs [72]. Since phosphorylation has been explicitly explored in the context of the Hippo signaling pathway, the fact that another PTM like acetylation could also regulate the activity of YAP and TAZ adds another layer to the importance of establishing a PTM atlas for MASLD. SIRT6 plays a vital role in protecting against fibrosis development by deacetylating YAP and TAZ at key lysine residues, like Lys102 for YAP1 and Lys39 for TAZ.
  - **Transforming growth factor-β and SMADs:** As MASLD progresses to MASH, accompanied by fibrosis, TGF-β stimulation acetylates Suppressor of Mothers against Decapentaplegic Homolog proteins (SMADs) SMAD2 and SMAD3 (Lys378) by p300/CBP [73]. Like the Hippo effectors above, SMADs are also intricately regulated by phosphorylation. The deacetylation of SMAD3 at Lys333 and Lys378 by SIRT6 negatively regulates their activity due to the TGF-β signaling pathway [74]. Moreover, both SMAD3 and SMAD2 are targets of SIRT6, which binds and deacetylates SMAD2 at Lys54 [75].
- **Mitochondrial Dysfunction and Oxidative Stress in MASLD**
  - **Citrate Synthase (CS):** Citrate synthase (CS), the first enzyme in the citrate acid cycle, catalyzes the conversion of oxaloacetate and acetyl-CoA to citrate. In *Escherichia coli*, CS is acetylated at multiple lysine residues [76]. Using a genetic code expansion strategy, the acetylation of Lys283 and Lys295 significantly affected CS activity. The acetylation of K283 doubled its activity, while the acetylation of K295 resulted in a 90% loss of activity. A dual-acetylated variant at K283 and K295 retained only 15% of the wild-type enzyme’s activity, indicating the dominant role of K295 acetylation in controlling CS activity [77].
  - **Mitochondrial trifunctional protein α-subunit (MTPα):** The acetylation of mitochondrial trifunctional protein α-subunit at lysine residues 350, 383, and 406 blocks its ubiquitylation and subsequent degradation, thereby promoting its stability and potentially influencing mitochondrial fatty acid oxidation. Studies using MTPα-deficient mice have shown the importance of this protein in mitochondrial function and lipid metabolism [78].
- **Proteostasis and ER Stress in MASLD**
  - **X-box binding protein 1 (XBP1):** XBP1, a key regulator of the unfolded protein response (UPR), plays a crucial role in maintaining proteostasis and alleviating ER stress in the liver. Studies have shown that liver-specific ablation of XBP1 disrupts the hepatic 12 h clock and promotes spontaneous NAFLD [79]. XBP1 also contributes to hepatocyte survival during ER stress by regulating the expression of IL-24, an anti-apoptotic protein. The disruption of IL-24 in XBP1-deficient mice increased cell death in response to liver injury [80]. These findings highlight the importance of XBP1 in maintaining ER homeostasis and protecting against liver damage in MASLD. XBP1s (the active spliced form of XBP1) is deacetylated by Sirt6 at lysine residues 257 and 297 [81]. This deacetylation promotes XBP1s protein degradation through the ubiquitin-proteasome system, ultimately protecting against ER-stress-induced hepatic steatosis. Conversely, the increased acetylation of XBP1s was observed in obese mice and those with hepatocyte-specific Sirt6 knockout, leading to increased hepatic steatosis.

**Table 3:** Acetylation and its impact on MASLD.

PTM	Target Protein	PTM Detection Methodology	Mechanism of Action	Impact on MASLD	Reference
Acetylation	SREBP1c (Lys289, Lys309)	Mass Spectrometry, Immunoblot	Stabilizes SREBP1c, promotes lipogenesis	Worsens steatosis	[50,51]
	LXR (Lys432)	Immunoblot	Modulates LXR transcriptional activity	Impacts lipid metabolism	[53]
	CREBH (Lys294)	Immunoblot	Promotes CREBH transcriptional activity	Impacts lipid metabolism	[57]
	ChREBP (Lys672)	Mass Spectrometry, Immunoblot	Promotes ChREBP transcriptional activity, increases lipogenesis	Worsens steatosis	[58,59,60]
	ACLY (Lys540, Lys546, Lys554)	Immunoblot	Stabilizes ACLY, promotes lipid synthesis	Worsens steatosis	[61]
	NF-κB/p65 (Lys310)	Mass Spectrometry	Enhances NF-κB transcriptional activity, promotes inflammation	Worsens inflammation	[70]
	YAP/TAZ	Immuno- precipitation	Promotes YAP/TAZ activity, promotes fibrosis	Worsens fibrosis	[64]
	SMAD2/3 (Lys378)	Immuno- precipitation	Promotes SMAD2/3 activity, promotes fibrosis	Worsens fibrosis	[65]
	CS (Lys283, Lys295)	Mass Spectrometry	Reduces CS activity, impairs mitochondrial function	Worsens mitochondrial dysfunction	[76,77]
	MTPα (Lys350, Lys383, Lys406)	Mass Spectrometry, Immunoblot	Stabilizes MTPα, promotes mitochondrial fatty acid oxidation	Ameliorates mitochondrial dysfunction	[69]
Deacetylation	SREBP1c (Lys289)	Immunoblot	Represses SREBP1c transcriptional activity	Ameliorates steatosis	[52]
	NF-κB p65	Mass Spectrometry	Inhibits NF-κB transcriptional activity, reduces inflammation	Ameliorates inflammation	[70]
	YAP/TAZ (Lys102, Lys39)	Immuno- precipitation	Inhibits YAP/TAZ activity, reduces fibrosis	Ameliorates fibrosis	[64]
	SMAD2/3 (Lys333, Lys378, Lys54)	Immuno- precipitation	Inhibits SMAD2/3 activity, reduces fibrosis	Ameliorates fibrosis	[66,67]
	XBP1s (Lys257, Lys297)	Mass Spectrometry, Immunoblot	Promotes XBP1s degradation, protects against ER stress	Ameliorates ER stress	[72]

### 4.4. Glycosylation

N-linked glycan alterations have been established to be signatures of liver diseases. While the pathogenesis of MASLD is multifactorial, emerging evidence suggests a crucial role for protein glycosylation in disease progression. Glycosylation, a complex post-translational modification involving the attachment of glycans (sugar molecules) to proteins, can significantly influence protein folding, stability, and function. The alteration of glycosylation patterns can itself derange liver functions, and screening for congenital disorders of glycosylation (CDG) should always be considered in patients with unexplained liver disease [82].

Glycosylation patterns are altered in various liver diseases, including MASLD. These alterations can affect protein functionality and contribute to disease progression (Table 4). The glycosylation of mammalian proteins is a non-template-based, tightly regulated biosynthetic process, the regulation of which depends on environmental, metabolic, genetic, and cellular factors. One of the key mechanisms is the change in the expression of glycosyltransferases, the enzymes responsible for catalyzing the transfer of sugar residues during glycosylation. For instance, a study utilizing mass spectrometry on 132 MASLD patients and 99 control subjects revealed that individuals with MASLD exhibited globally lower α2,3-sialylation compared to the control group [83]. Furthermore, non-fibrotic MASLD patients displayed higher levels of α2,3-sialylation compared to those with fibrotic MASLD. These findings suggest alterations in the N-glycan biosynthetic pathway, which could potentially serve as early diagnostic markers of fibrosis in MASLD. These findings, while promising, require further investigation and validation in larger cohorts. Higher levels of α2,3-sialylation in patients with MASLD were linked to galactosylation. It is important to note that changes in blood protein glycosylation could potentially replace liver biopsy for MASLD diagnosis.

The discovery of altered glycosylation patterns in MASLD has opened up new avenues for therapeutic intervention. Researchers are exploring the potential of targeting glycosylation pathways to modulate disease progression. For example, glycosylation inhibitors are being investigated for their ability to block the attachment of glycans to proteins, potentially restoring normal protein function and alleviating disease pathology. As glycosylation is considered a hallmark of cancer, interventions could investigate the potential of targeting oncogenic glycoforms and glycosyltransferases [84]. While this research is still in its early stages, it holds promise for developing novel therapies that can effectively target glycosylation anomalies in MASLD.

Abnormal glycosylation patterns have also been observed in other liver diseases, including alcoholic liver disease (with the decreased enzyme activities of mannosyltransferase and galactosyltransferase) [85]. This suggests that altered glycosylation may be a common feature in various liver pathologies, although the specific changes and their functional consequences may differ depending on the disease.

Another study involving human liver samples diagnosed with steatosis and nonalcoholic steatohepatitis (NASH) provided further evidence of N-linked glycosylation perturbations in NASH. The study revealed a downregulation of genes involved in N-glycan biosynthesis, including those responsible for precursor formation, the oligosaccharyltransferase complex, N-glycan quality control, trimming to the core, and extension from the core. These changes were associated with increased levels of unglycosylated uptake transporters (OATP1B1, OATP1B3, OATP2B1, NTCP) and the efflux transporter MRP2. Glycosylation directly impacts the transporters’ localization to the membrane and function, and reduced glycosylation can diminish their activity [86]. Importantly, reduced glycosylation of these transporters can affect drug disposition in NASH patients, with implications for treatment and management.

Recent spatial glycomics studies have shown early N-glycan alterations in Western diet models of MASLD using MALDI imaging mass spectrometry (IMS) [82]. The study found that the N-glycan profile of the liver changed significantly in response to the Western diet, with an increase in fucosylated N-glycans and a decrease in high mannose N-glycans. These changes were observed in specific liver regions, suggesting spatial heterogeneity in glycosylation patterns. The N-glycan changes were associated with early stages of liver injury, highlighting their potential as early diagnostic markers. The spatial nature of the N-glycan changes suggests that glycosylation patterns may be influenced by microenvironmental factors, such as nutrient availability, oxygen tension, and cell–cell interactions. Further research is needed to fully elucidate the role of glycosylation in MASLD and its potential as a therapeutic target.

- **Transporters**
  - **Multidrug Resistance-associated Protein 2 (MRP2):** MRP2, an efflux transporter, is affected by changes in glycosylation. Studies have reported an increase in the hemi-glycosylated form of MRP2 in NASH, along with the presence of its fully unglycosylated form [86,87]. These changes can potentially alter drug disposition in MASH patients.
- **Apolipoproteins**
  - **Apolipoprotein B-48 (ApoB-48):** Studies have shown that ApoB-48, a protein involved in lipid transport, can be modified by glycosylation. The aberrant glycosylation of ApoB, activated by N-acetylglucosaminyl transferase III (GnT-III), can inhibit ApoB assembly and block the synthesis and secretion of very-low-density lipoprotein (VLDL), leading to triglyceride accumulation in the liver, aiding in the progression to hepatocellular carcinoma (HCC) [88].
  - **Apolipoprotein B-100 (ApoB-100):** ApoB-100 is crucial for the assembly and secretion of very-low-density lipoprotein (VLDL) from the liver. It also acts as a ligand for LDL receptors, mediating the uptake of LDL cholesterol by cells. Changes in ApoB-100 glycosylation can disrupt these processes. For example, studies have shown that the increased glycation of ApoB-100 is associated with increased oxidative damage in patients with type 2 diabetes [89,90]. This oxidative damage can further impair LDL receptor binding and promote the accumulation of LDL cholesterol in the circulation, potentially contributing to MASLD progression. While the precise impact of glycosylation on ApoB-100’s function in MASLD requires further investigation, it is clear that this modification plays a role in its overall function and may be a potential therapeutic target.
  - **Nogo-B receptor (NgBR):** NgBR is involved in regulating lipid metabolism and insulin sensitivity [90]. Studies have shown that the expression of NgBR is decreased in the liver of individuals with obesity-associated type 2 diabetes and in mouse models of diabetes [90]. NgBR knockout in mouse hepatocytes resulted in increased blood glucose, insulin resistance, and β-cell loss [90]. Conversely, the overexpression of NgBR in the liver improved insulin sensitivity and reduced β-cell loss in diabetic mice. These findings suggest that NgBR may play a protective role in MASLD by improving insulin sensitivity and reducing hepatic lipid accumulation [91]. The loss of NgBR in endothelial cells leads to defects in the glycosylation of key endothelial proteins, including VEGFR2, VE-cadherin, and CD31, resulting in impaired vascular development [91]. It remains to be explored whether such a regulation of NgBR could potentially be a causal factor of MAFLD as well.
  - **NLRP3:** NLRP3 is a component of the NLRP3 inflammasome, a multiprotein complex that plays a crucial role in innate immunity and inflammation [92]. Activation of the NLRP3 inflammasome leads to the production of pro-inflammatory cytokines, such as IL-1β and IL-18, which contribute to liver inflammation and injury in MASLD [92]. A study reported that NLRP3 directly interacts with O-GlcNAc transferase (OGT), which stabilizes the protein, thus promoting MAFLD upon treatment with bisphenol A (BPA) [93]. While the exact glycosylation sites of NLRP3 have not been fully elucidated, studies suggest that glycosylation may influence its activity and contribute to the inflammatory response in MASLD [94].
  - **Aquaporin 9 (AQP9):** AQP9 is an aquaglyceroporin that facilitates the transport of water, glycerol, and other small solutes across cell membranes [95]. In the liver, AQP9 is involved in glycerol metabolism and may play a role in regulating hepatic lipid accumulation [95]. Studies have shown that AQP9 expression is altered in various liver diseases, including MASLD, and may contribute to disease progression [96]. Leptin-deficient (ob/ob) mice had significantly lower levels of glycosylated AQP9 protein in their livers compared to lean mice and the reduction was associated with impaired glycerol permeability in the liver [97]. While the specific glycosylation sites of AQP9 are not well-defined, glycosylation has been shown to increase the thermostability of human AQP10, a related aquaporin [98]. This suggests that glycosylation may also play a role in regulating the stability and function of AQP9 in MASLD.

**Table 4:** Glycosylation and its impact on MASLD.

PTM	Target Protein	PTM Detection Methodology	Mechanism of Action	Impact on MASLD	Reference
Glycosylation	MRP2	Immunoblot	Alters drug disposition	Impacts treatment efficacy	[76,77]
	ApoB-48	Metabolic Labeling	Inhibits ApoB assembly, blocks VLDL secretion	Worsens steatosis	[88]
	ApoB-100	Mass Spectrometry	Disrupts LDL receptor binding, promotes LDL cholesterol accumulation	Worsens steatosis	[78,79]
	NLRP3	Immunoblot	Influences NLRP3 activity, promotes inflammation	Worsens inflammation	[81,82,83]
	AQP9	Immunoblot	Regulates glycerol metabolism, impacts hepatic lipid accumulation	Impacts lipid metabolism	[84,85,86,87]

## 5. Conclusions: PTM Memory Holds the Key to Understanding Disease Pathology

This review underscores the critical role of post-translational modifications (PTMs) in the onset and progression of MASLD and MASH, emphasizing their potential as therapeutic targets. PTMs are not only vital for cellular homeostasis but also represent dynamic regulators of protein function and stability. Their crosstalk—both intra- and inter-PTM—contributes significantly to cellular processes, with disruptions often leading to the destabilization of protein–protein interactions and structural integrity. The concept of ***PTM* “*memory*”** is emerging as a pivotal factor in understanding chronic disease pathology. PTM memory refers to the ability of cells to retain epigenetic and molecular signatures of previous stress or environmental changes, which subsequently influence cellular behavior. Aberrant PTM memory may perpetuate pathological states, such as chronic inflammation or fibrosis, by sustaining detrimental modifications or failing to reset to a healthy baseline. Addressing these anomalies through the targeted modulation of PTM memory could offer transformative therapeutic opportunities.

Emerging evidence suggests that PTMs play a crucial role in maintaining pathological memory in hepatocytes, Kupffer cells, and hepatic stellate cells, contributing to disease progression even after the initial insult (e.g., high-fat diet, alcohol exposure) is removed.

### 5.1. Metabolic Memory in NAFLD: Persistent Acetylation and O-GlcNAcylation

#### 5.1.1. Acetylation of PGC-1α in Hepatic Lipid Metabolism

Peroxisome proliferator-activated receptor γ coactivator 1-α (PGC-1α) is a key regulator of mitochondrial function and fatty acid oxidation. In NAFLD, high-fat diet exposure leads to the persistent acetylation of PGC-1α, reducing its activity and impairing fatty acid oxidation, promoting lipid accumulation in hepatocytes. Even after dietary normalization, deacetylation remains incomplete, leading to continued mitochondrial dysfunction and a metabolic memory of hepatic steatosis [99].

**Key PTMs involved**: The acetylation of PGC-1α at K778 is regulated by SIRT1.

#### 5.1.2. O-GlcNAcylation of ChREBP and Lipogenic Memory

ChREBP is a master transcription factor that regulates hepatic lipogenesis. Chronic glucose and fructose exposure lead to the O-GlcNAcylation of ChREBP, increasing its stability and promoting de novo lipogenesis. This modification persists even after glucose normalization, maintaining excessive lipid accumulation in the liver [100].

**Key PTMs involved**: The O-GlcNAcylation of ChREBP at S626, which is regulated by OGT.

### 5.2. Inflammatory Memory in NASH: The Phosphorylation and SUMOylation of NF-κB

#### 5.2.1. Phosphorylation of NF-κB and Kupffer Cell Memory

Kupffer cells (liver-resident macrophages) exhibit trained immunity in NASH, characterized by the persistent activation of NF-κB even after inflammatory stimuli subside. The phosphorylation of NF-κB (p65 subunit) at S536 leads to prolonged nuclear retention, sustaining pro-inflammatory cytokine expression (e.g., TNF-α, IL-6). This creates a post-translational inflammatory memory, predisposing the liver to chronic inflammation and fibrosis [101].

**Key PTMs involved**: The phosphorylation of NF-κB (p65) at S536 by IKKβ.

#### 5.2.2. SUMOylation of NF-κB Inhibitors and Chronic Inflammation

Under normal conditions, IκBα (an NF-κB inhibitor) suppresses inflammatory signaling. In NASH, the SUMOylation of IκBα reduces its degradation, leading to a paradoxically prolonged NF-κB activation, sustaining inflammation. This PTM-mediated dysregulation persists even after dietary or pharmacological interventions, explaining why the resolution of NASH is difficult [102].

**Key PTMs involved**: The SUMOylation of IκBα at K21, reducing degradation.

### 5.3. Fibrotic Memory in Liver Disease: Persistent Collagen Production via Methylation

#### 5.3.1. Methylation of H3K4 in Hepatic Stellate Cells (HSCs) and Persistent Fibrosis 

Hepatic stellate cells (HSCs) are the key drivers of liver fibrosis, producing excess extracellular matrix (ECM) proteins like collagen-1 (COL1A1). Even after fibrotic stimuli (e.g., alcohol, obesity) are removed, HSCs retain a fibrotic phenotype due to persistent histone H3K4 methylation at the COL1A1 promoter.

This epigenetic PTM-based memory ensures that HSCs remain primed for fibrosis, contributing to fibrosis recurrence in patients with NAFLD or ALD [103].

**Key PTMs involved**: H3K4 trimethylation (H3K4me3) at the COL1A1 promoter, mediated by SETD1A (histone methyltransferase).

#### 5.3.2. Phosphorylation of SMAD3 in TGF-β Signaling and Fibrotic Persistence

The TGF-β/SMAD3 pathway is central to liver fibrosis. In fibrotic livers, SMAD3 is persistently phosphorylated at S423/S425, maintaining excessive ECM production even after the resolution of the injury. This PTM-based memory keeps HSCs in an activated state, making fibrosis difficult to reverse [104].

**Key PTMs involved**: The phosphorylation of SMAD3 at S423/S425, mediated by TGF-β receptor kinases.

Future therapies might focus on enhancing biologically beneficial PTMs while suppressing harmful ones. A pertinent example is the phosphorylation of VPS4A at Ser95 and Ser97, which has recently been shown to regulate lipophagy—a selective autophagy process targeting lipid droplets [40]. The dysregulation of VPS4A phosphorylation can impair lipophagy, contributing to lipid accumulation and metabolic dysfunction. Therapeutic approaches aimed at restoring optimal VPS4A phosphorylation levels could not only improve lipophagy efficiency but also alleviate lipid-induced hepatotoxicity, presenting a novel strategy for MASLD treatment.

## 6. Future Perspectives

The role of PTM memory in disease pathology represents a compelling frontier in our understanding of the chronicity and persistence of disorders like MASLD. PTM memory can perpetuate maladaptive cellular states, acting as a molecular “bookmark” that predisposes cells to disease recurrence or progression. In MASLD, aberrant PTM memory may sustain pathways that favor lipid accumulation, oxidative stress, or inflammatory signaling, thereby exacerbating liver damage.

Deciphering these molecular imprints through high-resolution proteomics could unveil critical insights into disease progression and therapeutic resistance. The ability to “reset” pathological PTM memory or replace it with healthy molecular states could revolutionize therapeutic strategies. For instance, by targeting VPS4A phosphorylation anomalies, researchers could potentially modulate lipophagy to reverse lipid accumulation and restore hepatic homeostasis.

Moreover, studying PTM crosstalk and memory may provide a framework for precision medicine. The identification of PTM-based biomarkers could allow for the stratification of patients according to their disease severity, drug response, or risk of progression. By targeting PTM anomalies, including those embedded in PTM memory, novel therapeutics could simultaneously address disease symptoms and underlying molecular dysfunctions.

Looking ahead, integrating advanced proteomic techniques with computational modeling and machine learning will be crucial for mapping the complex PTM networks that underpin MASLD and related diseases. These efforts will not only enhance our understanding of PTM memory but also pave the way for groundbreaking therapies that redefine the management of chronic diseases like MASLD.

## Data Availability

No new data were created or analyzed in this study. Data sharing is not applicable to this article.
